# Pyramidal Angulation of the Ascending Aortic Graft: Recurrent Emboli After Type A Repair

**DOI:** 10.1016/j.jaccas.2026.108131

**Published:** 2026-06-10

**Authors:** Naomi Timmermans, Kayan Lam, Pieter-Jan Vlaar, Thomas van Brakel

**Affiliations:** a Department of Cardiothoracic Surgery, University Medical Center Utrecht, Utrecht, the Netherlands; b Department of Cardiothoracic Surgery, Catharina Hospital Eindhoven, Eindhoven, the Netherlands

**Keywords:** anticoagulation, aortic dissection, ascending aorta graft, embolic stroke, graft kinking, redo surgery

## Abstract

**Background:**

We report a unique case of recurrent cerebrovascular events under adequate anticoagulation, in the absence of a visible thrombus, due to a severely kinked ascending aortic graft after type A dissection repair.

**Case Summary:**

A 70-year-old man developed 4 transient ischemic attacks and 3 ischemic strokes over 4 years despite therapeutic rivaroxaban levels and prior antiplatelet therapy. Imaging revealed a kinked ascending graft without thrombus. High-risk redo surgery was pursued to correct graft geometry, after which no further embolic events occurred during early follow-up (5 months).

**Discussion:**

This case addresses the clinical dilemma whether a visible thrombus is required to justify redo surgery after type A repair, especially when recurrent embolism occurs under maximal medical therapy.

**Take-Home Message:**

Surgical correction of graft kinking may be indicated—even in the absence of a thrombus—when embolic risk persists under optimal anticoagulation.

## Visual Summary

**Figure:**
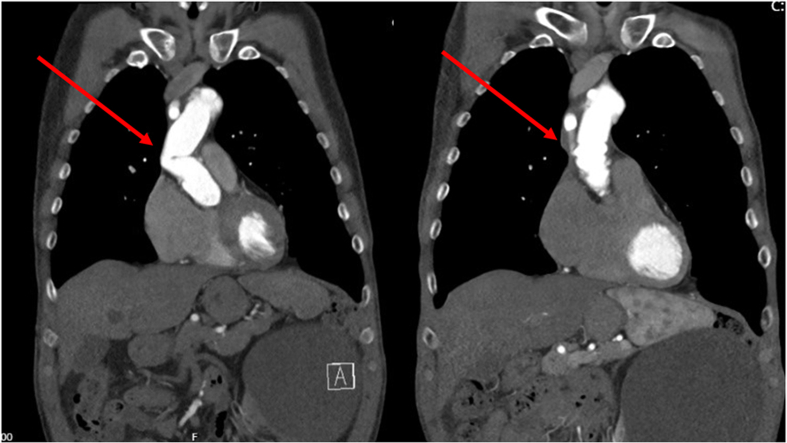

**Visual Summary:**
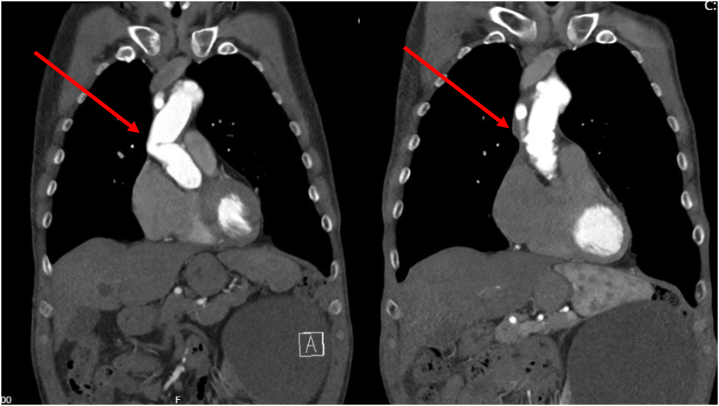
Imaging Timeline The red arrow indicates the ascending aortic graft before and after correction.

## History of Presentation

A 70-year-old man presented with recurrent cerebrovascular events under antiplatelet monotherapy and direct oral anticoagulation therapy (DOAC)—3 ischemic strokes and 4 transient ischemic attacks—over the course of 4 years after emergency ascending aortic replacement and zone 1 arch reconstruction with reimplantation of the brachiocephalic trunk for acute type A dissection in 2018.

**Take-Home Messages**

• In cases of recurrent embolism under therapeutic dose oral anticoagulation, a severely kinked graft may necessitate redo surgery—even in the absence of a visible thrombus.
• A precise surgical technique and graft alignment are essential in preventing postoperative embolic complications.

## Past Medical History

In 2011, there was a deep vein thrombosis for which 3 months of therapeutic anticoagulation was required. A recurrent deep vein thrombosis in 2021 led to an indication for lifelong DOAC treatment.

## Investigations

A comprehensive diagnostic evaluation was conducted in a multidisciplinary setting.

Neurologic assessment included carotid duplex ultrasonography, which revealed no abnormalities.

Cardiac evaluation by the cardiology team included transthoracic echocardiography and a 7-day Holter monitor, neither of which demonstrated a cardiac embolic source.

Internal medicine specialists performed an extensive laboratory work-up for coagulation disorders and vasculitis, all yielding normal results. The patient was a normal CYP2C19 metabolizer and had therapeutic rivaroxaban plasma levels on multiple occasions. Also, no evidence was found for malignancies on multiple computed tomography (CT) scans.

Given the recurrent embolic events and inconclusive findings, the case was referred to an academic center for a neurologic second opinion. Electrocardiogram-gated CT angiography with 3-dimensional reconstructions revealed a severe kink in the aortic graft prosthesis, raising strong suspicion that this geometric abnormality was the underlying etiology of the recurrent embolic phenomena, although there was no evidence of an intraluminal thrombus (Figure 1).

**Figure 1:**
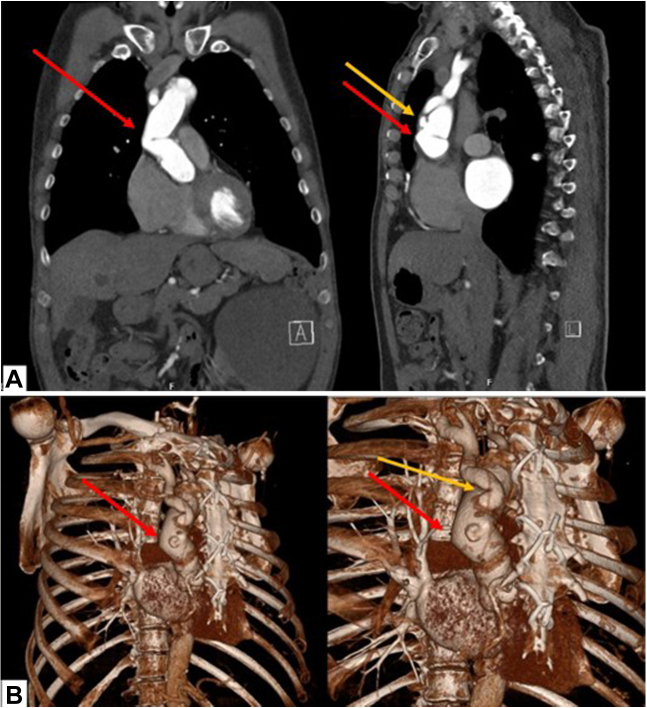
Imaging Before Correction of the Kinked Graft (A) Frontal and sagittal CT reconstruction after 6 years of follow-up. A marked kink due to elongation of the ascending aortic graft is visible. Also kinking of the anteflow leg connected with the brachiocephalic trunk. (B) CT 3-dimensional reconstruction after 6 years of follow-up. CT = computed tomography. The red arrow indicates kinking in the ascending aortic graft. The yellow arrow indicates kinking of the first aortic arch branch graft.

## Management

Despite extensive neurologic evaluation and adequate anticoagulation therapy with a DOAC, events continued to occur. Previously, in one of the last episodes of cerebral ischemic events, the patient also experienced hemorrhagic transformation; therefore, antiplatelet therapy in combination with DOAC was not favorable, which limited the intensification of treatment. Thus, medical therapy was considered maximal with DOAC.

Although the absence of a thrombus on CT angiography complicates the decision for redo surgery, the graft kinking was considered the most likely source of thromboembolism under continuous optimal anticoagulation.

Redo surgery was performed with replacement of the ascending graft by a new shortened single-branch prosthesis, thereby ensuring that there was no kinking left. Zone 1 arch repair was conducted with continuous flow-controlled perfusion via the right subclavian and femoral arteries. During surgery, no thrombi were found in the aortic prosthesis at the site of the kink.

DOAC was recontinued after the surgery because of the recurrence of stroke preoperatively.

## Outcome and Follow-Up

The postoperative course was uneventful, and the patient was discharged on postoperative day 5. Follow-up CT angiography at 6 weeks demonstrated correct alignment of the graft without residual kinking (Figure 2). During initial follow-up, no further ischemic events occurred. Long-term follow-up is needed to confirm durability of this result.

**Figure 2:**
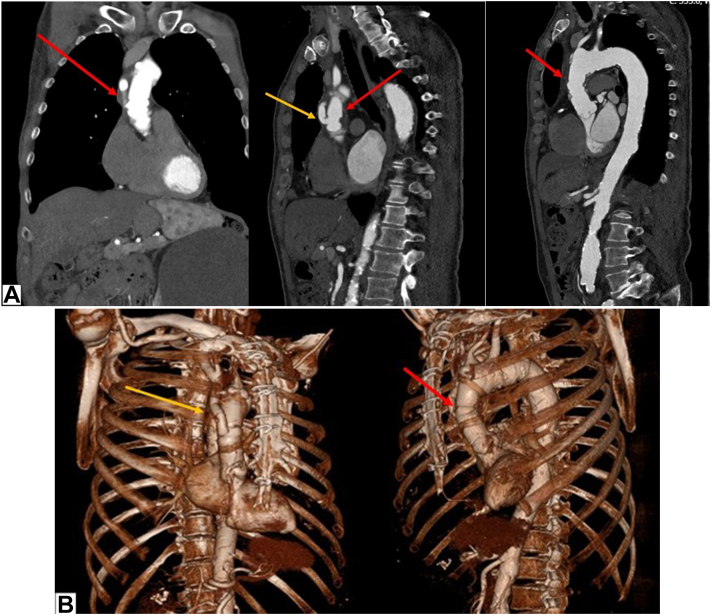
Imaging After Correction of the Kinked Graft (A) Frontal and sagittal CT reconstruction after redo surgery. There is no more kinking of the ascending aortic graft or the brachiocephalic trunk. (B) CT 3-dimensional reconstruction after correction. CT = computed tomography. The red arrow indicates the corrected ascending aortic graft. The yellow arrow indicates the corrected aortic arch branch.

## Discussion

Kinking of ascending aortic prosthetic grafts is rare but may result in hemodynamic compromise and thromboembolic complications. In the case described by Hasami et al,^1^ a severe graft kink with associated mural thrombus led to a cerebrovascular accident and ultimately redo surgery. Our case differs in that recurrent embolic events occurred despite therapeutic anticoagulation, but without visible thrombus on imaging—making the decision for high-risk redo surgery particularly challenging.

These cases raise 3 central issues: 1) when to consider redo surgery; 2) the role and limitations of postoperative anticoagulation; and 3) how to prevent technical pitfalls such as “pyramids” during the index operation.

Redo aortic surgery carries significant risk, and indication setting must be carefully individualized. Traditionally, visible thrombus, anastomotic failure, or aneurysmal dilation is an accepted trigger. Our case and the case of Hasami et al^1^, ^2^ expand this paradigm: recurrent embolism under optimal medical therapy, in the setting of severe prosthetic kinking, is itself a surgical indication—even without visible thrombus. In both patients, correction of graft geometry eliminated embolic recurrence.^1^^,^^2^ This supports incorporating graft configuration into decision-making, particularly in the context of unexplained cerebrovascular events.

There are no established guidelines for postoperative antithrombotic therapy after ascending aortic repair. Most centers use single antiplatelet therapy unless another indication exists. Both cases highlight the challenges when embolic events recur despite anticoagulation. In the case of Hasami et al,^1^ a thrombus was documented at the kink, and anticoagulation was escalated. In the current case, repeated events occurred under therapeutic DOAC therapy, with bleeding complications preventing escalation to combined therapy. These experiences emphasize that when disturbed flow due to graft kinking persists, no medical regimen can fully mitigate embolic risk. Antithrombotic therapy should be optimized, but specialists must recognize when surgery becomes the more definitive solution.^1^, ^2^, ^3^

Both cases demonstrate that a precise surgical technique during the index operation is essential to prevent later complications. A particularly important pitfall is the so-called pyramid phenomenon, which arises when an overly long or continuous prosthesis is implanted across the sharp angle between the aortic root and arch. This configuration predisposes the graft to kinking and subsequent flow disturbances. To avoid this, surgeons should carefully measure and tailor graft length, consider the use of separate graft segments at anatomical bends to minimize redundancy, and ensure that the prosthesis respects the natural curvature of the aorta. Intraoperative tools such as transesophageal echocardiography or angioscopy can provide additional confirmation of proper alignment.^4^ By preventing pyramids at the time of surgery, the risk of severe graft kinking and the need for high-risk redo operations can be significantly reduced.^4^^,^^5^

Taken together, our experience supports a pragmatic approach: prevent graft kinking at the index operation; in patients with recurrent ischemic events despite therapeutic anticoagulation and severe graft angulation, a visible thrombus is not required to consider anatomical correction once alternative sources are excluded. Management should be case-by-case and patient-tailored, weighing the presence/absence of a thrombus, event profile (transient ischemic attack vs stroke), degree/location of angulation, response or intolerance to antithrombotics, operative risk, and patient preference.

## Conclusions

Severe graft kinking can be a source of embolic events even without a visible thrombus, and high-risk surgical correction may be the definitive solution when medical therapy fails. Recognition of this scenario and preventive surgical strategies are key to improving outcomes and reduce the incidence of this under-recognized complication.

## Funding Support and Author Disclosures

The authors have reported that they have no relationships relevant to the contents of this paper to disclose.

## References

[1] Hasami N.A., Geuzebroek G.S.C., Smith T., Heijmen R.H. (2025). Severely kinked ascending aorta prosthetic graft causing thromboembolism. JACC Case Rep.

[2] Tamura Y., Funaki T., Yamamoto K., Kodera K. (2022). Hemolytic anemia caused by kinked graft 6 months after central repair of acute aortic dissection. J Surg Case Rep.

[3] Feng X., Zhao Y., Zhang H., Li Y., Zhi X., Liu J. (2024). Clinical study of reoperation for acute type A aortic dissection. Front Cardiovasc Med.

[4] Kaleda V.I., McCormack D.J. (2021). How to deal with graft kinking in ascending aortic surgery. CTSNet. https://www.ctsnet.org/article/how-deal-graft-kinking-ascending-aortic-surgery.

[5] Isselbacher E.M., Preventza O., Black J.H. (2022). Imaging and surveillance of chronic aortic dissection: a scientific statement from the American Heart Association. Circ Cardiovasc Imaging.

